# Outcomes following Placement and Removal of Transvaginal Cerclage in at Risk Pregnancies: A Single Center Experience

**DOI:** 10.1155/2022/4277451

**Published:** 2022-07-13

**Authors:** Henry Adekola, Jennifer Addo, Elizabeth Ramsey Unal, Emma James, Vivek Prakash, Robert Abrams

**Affiliations:** ^1^Department of Maternal-Fetal Medicine, Southern Illinois University School of Medicine, USA; ^2^Department of Obstetrics and Gynecology, Southern Illinois University School of Medicine, USA; ^3^Center for Clinical Research, Southern Illinois University School of Medicine, USA

## Abstract

**Objective:**

The objective of this study was to estimate the time between removal of cerclage and delivery, stratified by indication for cerclage placement (elective or non-elective). Additionally, delivery within 72 hours after cerclage removal was compared between elective and non-elective cerclage placement, as well as between ultrasound-indicated and physical examination-indicated cerclage placement.

**Design:**

A single-center retrospective cohort study. *Participants/Materials*, *Setting, and Methods.* Clinical information of 72 pregnant women who underwent transvaginal cerclage over a 4-year period was obtained. Comparisons were made between elective (history-indicated) and non-elective (ultrasound or physical examination-indicated) cerclage placement. Comparisons were also made between physical examination-indicated and ultrasound-indicated cerclage.

**Results:**

Compared to those who had a non-elective cerclage, women undergoing elective cerclage were more likely to have history of cervical treatment (44% vs. 15%, *p* = 0.02), and spontaneous preterm delivery (92% vs. 61%, *p* = 0.003). There was no difference in the rate of delivery ≤72 hours following cerclage removal between women who had elective cerclage and those who had non-elective cerclage (46% vs. 58%, *p* = 0.47). Women who had an elective cerclage were more likely to have elective cerclage removal ≥36 weeks (71.8% vs. 39.4%, *p* = 0.01), compared to those who had undergone non-elective cerclage. The rate of delivery ≤72 hours following removal of cerclage was greater in women who had a physical examination-indicated cerclage compared to women who had ultrasound-indicated cerclage (80% vs. 39%, *p* = 0.04). Among women who had an elective cerclage, there was no difference in the rate of delivery at ≤72 hours between those who had elective cerclage removal at 36 weeks compared to those electively removed at 37 weeks (31% vs. 58%, *p* = 0.30). No complications such as fetal demise, iatrogenic amniotic membrane rupture, hemorrhage, or cervical laceration were reported within this cohort.

**Conclusion:**

Cerclage indication should be considered prior to scheduling elective cerclage removal. Women who had an elective cerclage are most likely to get it electively removed at 36 weeks compared to their counterparts who had a non-elective cerclage. Furthermore, women who had a physical examination-indicated cerclage are most likely to deliver within 72 hours of cerclage removal.

## 1. Introduction

Preterm birth is a significant contributor to neonatal morbidity and mortality, death in children aged 5 years or under, as well as longstanding disability in the USA [1–4]. Nearly 1 in 10 births occur before 37 weeks [5] and preterm birth has been estimated to cost $26 billion dollars in annual health care expenditure [6] .

Cervical cerclage is one tool available to mitigate the risk of preterm birth in women with history of cervical insufficiency (history-indicated), asymptomatic cervical dilatation in the mid-trimester (physical examination-indicated), and asymptomatic short cervix (<25 mm) in the mid-trimester with history of spontaneous preterm birth (ultrasound-indicated) [7]. Enakpene and colleagues also demonstrated that in women without a history of spontaneous birth but with extremely short cervix (≤ 10 mm), cervical cerclage plus vaginal progesterone significantly decreased overall spontaneous preterm birth rates, prolonged pregnancy latency by 2-fold, and decreased overall neonatal morbidity and mortality [8].

The final common pathway for the various etiologies of preterm parturition syndrome leads to shortening and dilatation of the cervix, which in turn leads to fetal inflammatory response and ultimately culminates in preterm delivery [9, 10]. A long, closed cervix provides both mechanical support as well as an immunological barrier to protect the fetus from pathogens within the vaginal microbiome [11]. The cervical mucus plug has been demonstrated to have antibacterial properties [12–14]. Thus, a cervical cerclage, in addition to providing physical support to the cervix, may also function to augment the immunological barrier by retention of the cervical mucus plug.

Multiple experts have demonstrated the benefits of cervical cerclage in the management of pregnancies at risk for spontaneous preterm birth [8, 15–19]. It is the authors' opinion that the outcome of the recent PROLONG study [20] may lead to increased cervical length surveillance and thus increased utilization of cerclage in pregnant women at risk of spontaneous preterm birth. Hence, there is a need for continued critical assessment of outcomes following cerclage placement.

The objective of this study was to estimate the time between removal of cerclage and delivery, stratified by indication for cerclage placement. Additionally, delivery within 72 hours after cerclage removal was compared between groups.

## 2. Material and Methods

### 2.1. Study Design and Data Collection

We conducted a retrospective cohort study in the Department of Obstetrics and Gynecology of HSHS St. John's Hospital, which is affiliated with Southern Illinois University School of Medicine, Springfield, IL, USA.

The Institutional Review Boards of Southern Illinois University School of Medicine and HSHS St. John's Hospital approved this study (IRB REF No.20-275; February 2020). Clinical information was obtained from medical records of pregnant women with transvaginal cerclage performed between January 1, 2015, and December 31, 2019.

History-indicated cerclage was performed at 12-15 weeks for a history consistent with cervical insufficiency [7]. Ultrasound-indicated cerclage was placed at 16-23 6/7 weeks if the woman had a prior spontaneous preterm birth and a current cervical length of <25 mm [7]. Physical examination-indicated cerclage was indicated on the discovery of painless cervical effacement and dilatation, in the absence of infection symptomatology or vaginal bleeding [7].

Exclusion criteria included multiple gestation, fetal anomaly, fetal aneuploidy, painful uterine contractions, suspected clinical chorioamnionitis, and fetal demise.

Cerclage was performed with McDonald or Shirodkar technique using Mersilene 5 mm tape™ or Ethibond™ (Ethicon, Somerville, NJ, USA). All procedures were performed under spinal anesthesia with indomethacin administered for 3 days post procedure, unless otherwise indicated.

Elective cerclage removal was performed between 36 and 37 weeks in asymptomatic women, depending on the preference of the clinician. Earlier (non-elective) removal was indicated when imminent delivery was suspected, prelabor rupture of membranes occurred in periviable gestation, or fetal demise was diagnosed.

## 3. Definitions

### 3.1. Elective Cerclage Placement

Cerclage placement performed at 12-15 weeks due to history consistent with cervical insufficiency (history-indicated).

### 3.2. Non-elective Cerclage Placement

Cerclage placement performed at 16-23 6/7 weeks due to short cervical length on ultrasound (ultrasound-indicated) or painless cervical dilatation on physical examination (physical examination-indicated).

### 3.3. Elective Cerclage Removal

Scheduled cerclage removal at 36-37 weeks (in the absence of labor or preterm prelabor rupture of membrane) or at the time of scheduled cesarean delivery.

### 3.4. Non-elective Cerclage Removal

Cerclage removed due to onset of spontaneous preterm labor, prelabor rupture of membranes in previable gestational age, fetal demise, or any other condition in which imminent delivery is predicted to occur.

### 3.5. History of Cervical Treatment

Cervical treatments may include loop electrosurgical excision procedure of the cervix, cold-knife cone biopsy of the cervix, and cervical trachelectomy.

### 3.6. Statistical Analysis

In all tables, continuous variables are presented as mean ± standard deviation, while categorical variables are presented as *n* (%). Comparison of continuous variables was performed using Student *t* test. Due to clustering around zero, time between cerclage removal and delivery was tested with a likelihood ratio test of negative binomial regressions. Chi square test was used to compare categorical variables. For all tests, a *P* value < 0.05 was considered statistically significant. Statistical analysis was performed with R version 3.6.3(R Foundation for Statistical Computing, Vienna, Austria).

## 4. Results

A total of 75 transvaginal cerclage procedures were performed during the study period. Of these, 3 women were excluded from the study due to inability to obtain complete medical records. Thirty-nine (54.2%) women had an elective cerclage (history indicated cerclage), and 33(45.8%) women underwent non-elective cerclage (15 [20%] physical examination-indicated and 18 [25.8%] ultrasound indicated).

Comparison of maternal demographic data and outcome variables between women treated with elective cerclage and those treated with non-elective cerclage is shown in Table 1. There was no difference between groups in race (*P* = 0.85), maternal age (*P* = 0.71), gravidity (*P* = 0.99), tobacco use (*P* = 0.83), or marijuana use (*P* = 0.12). The rates of prior cervical treatment (44% vs. 15%; *P* = 0.02) and history of preterm birth (92% vs. 61%; *P* = 0.003) were significantly higher and gestational age at cerclage placement significantly earlier (15.02 ± 0.34 weeks vs. 20.51 ± 0.43 weeks; *P* < 0.01) in women who underwent elective cerclage, compared to their counterparts who had non-elective cerclage placement. There was no difference between groups regarding gestational age at cerclage removal (*P* = 0.36), gestational age at delivery (*P* = 0.33), delivery within 72 hours after cerclage removal (*P* = 0.47), and time interval between cerclage removal and delivery (8.33 ± 1.67 days vs. 6.91 ± 1.59 days; *P* = 0.64). However, a significantly greater proportion of women in the elective cerclage group had elective cerclage removal at ≥36 weeks compared to their counterparts who underwent a non-elective cerclage placement (71.8% vs. 39.4%, *P* = 0.01).

Comparison of outcomes between women treated with physical examination-indicated cerclage versus ultrasound-indicated cerclage is shown in Table 2. There was no difference in gestational age at cerclage placement (21.33 ± 0.37 weeks vs. 19.83 ± 0.69 weeks, P = 0.07), gestational age at cerclage removal (33.83 ± 0.86 weeks vs. 33.62 ± 1.11 weeks, *P* = 0.88), rate of non-elective cerclage removal (7[47%] vs. 7 [39%] *P* = 0.78), or gestational age at delivery (34.38 ± 0.97 vs. 34.99 ± 1.29 weeks, *P* = 0.71) between women who had physical examination-indicated cerclage and women who had ultrasound-indicated cerclage. There was a significantly higher rate of delivery within 72 hours of cerclage removal (12 [80%] vs. 7 [39%]; *P* = 0.04) in women who had undergone physical examination indicated cerclage compared to women with ultrasound-indicated cerclage. Although there was a shorter time interval between cerclage removal and delivery in women who had a physical examination indicated cerclage compared to women who had ultrasound indicated cerclage (3.67 ± 1.86 days vs. 9.61 ± 2.33 days; *P* = 0.14), this was not statistically significant.

A subgroup analysis within patients who had both elective cerclage placement and elective cerclage removal, comparing those in whom cerclage was removed at 36 weeks with their counterparts whose cerclage was removed at 37 weeks, is shown in Table 3. There was no difference between groups regarding gestational age at cerclage placement (14.95 ± 0.40 weeks vs. 15.37 ± 0.82 weeks, *P* = 0.65) or delivery within 72 hours of cerclage removal (5 [31%] vs. 7 [58%], *P* = 0.30). While women who had elective cerclage removal at 36 weeks had a longer time interval between cerclage removal and delivery when compared to women who had elective cerclage removal at 37 weeks, this was not statistically significant (9.38 ± 2.12 days vs. 3.83 ± 1.43 days; *P* = 0.13).

There were no reported complications including fetal demise, iatrogenic rupture of amniotic membrane, bleeding and/or cervical laceration at the time of cerclage placement or cerclage removal.

## 5. Discussion

### 5.1. Summary of Study Findings

The principal findings of this study are as follows: A greater proportion of women with history of cervical treatment and spontaneous preterm birth were observed among those who had elective cerclage placement compared with those who had non-elective cerclage placement.

There was no difference in either the rate of delivery ≤72 hours following removal of cerclage or the total time interval between cerclage removal and delivery between women who had elective cerclage placement and those who had non-elective cerclage placement. The delivery rate ≤ 72 hours following removal of cerclage was greater in women who had a physical examination-indicated cerclage compared to those who had an ultrasound-indicated cerclage. A similar trend was observed in the total time interval between removal of cerclage and delivery, though this was not statistically significant. A greater proportion of patients undergoing elective cerclage compared to those who underwent non-elective cerclage had elective cerclage removal at 36 weeks or greater.

In women who had a history-indicated(elective) cerclage, there was no difference in the rate of delivery ≤72 hours between those who had their cerclage electively removed at 36 weeks compared to women who had theirs removed at 37 weeks. In this cohort of 72 patients, no complications such as fetal demise, iatrogenic amniotic membrane rupture, hemorrhage, or cervical laceration following cerclage placement or removal were reported.

### 5.2. Interpretation of Findings and Comparison with Published Literature

Our finding that women undergoing elective cerclage were more likely to have a history of prior spontaneous preterm birth is expected and consistent with multiple studies [21–23]. Similar to Guzman et al. [24], we also observed a higher proportion of women with history of cervical treatment among women who had an elective cerclage compared to a non-elective indication for cerclage placement. This differs from the findings by Alabi-Isama et al. [23] that demonstrated that a higher proportion of prior cervical treatment in women undergoing ultrasound indicated cerclage as compared to their counterparts undergoing history indicated (elective) cerclage. Other investigators have found no difference between aforementioned groups [21, 22].

While there was no difference in gestational age at cerclage removal between elective and non-elective cerclage placement, it should be noted that the average gestational age at cerclage removal was 34.72 weeks and 33.72 weeks, respectively. This may be due to the fact that almost half of the women who had elective cerclage in our study had a history of prior cervical treatment and over 75% of the cohort had a history of spontaneous preterm birth. It should be noted that unlike other studies [21–23], we did not exclude women delivering before 36 weeks gestational age from this cohort. This is important as we evaluated outcomes in all individuals who received cerclage.

It may not be surprising that a greater proportion of women who an elective cerclage compared to those who had a non-elective cerclage had elective cerclage removal at ≥36 weeks. Elective cerclage is almost always performed on a closed and uneffaced cervix. Hence, elective cerclage placement is most likely performed in the absence of intraamniotic inflammation and/or infection. It may also not be surprising that within the subgroup of patients who had both elective cerclage placement and removal, there was no difference in the proportion of women who delivered within 72 hours of cerclage removal or total time interval between removal of cervical cerclage and delivery.

Conversely, a greater proportion of women who underwent physical exam-indicated cerclage delivered at or less than 72 hours following removal of cerclage compared to their counterpart undergoing ultrasound indicated cerclage. A similar trend was noted when the total time interval between cerclage removal and delivery was compared, though this comparison did not reach statistical significance. The rate of intraamniotic inflammation/infection has been shown to be as high as 50% in women with mid-trimester painless cervical dilatation [25] and about 9% in mid-trimester asymptomatic short cervices <15 mm [26]. While amniocentesis was not performed prior to non-elective cerclage placement in our patients, we hypothesized that there must have been a higher rate of intraamniotic inflammation/infection in women who had a physical examination-indicated cerclage compared to women who had ultrasound indicated cerclage. It should be noted that experts have demonstrated a good prognosis when painless cervical dilatation is managed with cerclage in the absence of intraamniotic infection/inflammation as confirmed by amniocentesis [25].

### 5.3. Clinical Implications of Study

Complications such as cervical laceration and uterine rupture have been described in women laboring with cerclage in place. Therefore, most providers aim to remove cerclage at 36 to 37 weeks, to minimize the risk of laboring with a cerclage in situ. Some providers elect to remove the stitch at 37 weeks, as the goal of cerclage placement itself is to prevent delivery prior to 37 weeks. Our study, similar to other investigators [23], demonstrates that term delivery is still achievable regardless of whether a cervical cerclage is electively removed at 36 or 37 weeks gestational age, especially if said cerclage was placed as a history-indicated procedure.

Based on our findings, we propose that indication for cerclage placement should be considered prior to elective cerclage removal. Those women who had history-indicated (elective) cerclage placement can have their cerclage electively removed at 36 weeks or 37 weeks depending on provider and/or patient preference. Hence, they can be counseled that term delivery is likely. Conversely, significantly fewer women who had non-elective cerclage had elective cerclage removal at 36 weeks or greater. Therefore, it can be inferred that while a non-elective cerclage may prolong pregnancy beyond viability, a significant proportion of these women will deliver preterm. Furthermore, for those who had a non-elective cerclage, elective removal of cerclage at 37 weeks should be considered especially if cerclage placement was indicated due to physical examination. A higher rate of delivery at 72 hours or less following cerclage removal in physical examination-indicated cerclage may support this. This should also be considered in women who an ultrasound-indicated cerclage, as nearly 40% delivered within 72 hours of cerclage removal.

### 5.4. Strengths and Limitations of Study

Unlike other studies that excluded women delivering before 36 weeks, we included all patients who underwent cerclage, regardless of timing of delivery. This study is also unique as a comparison of outcomes stratified by all 3 indications for cerclage placement was performed.

As this was a retrospective study, the risk of bias due to case selection is a recognized limitation. In addition, none of the women undergoing non-elective cerclage had an amniocentesis which may have shed light onto the reasons behind for a higher rate of delivery at 72 hours or less following cerclage removal in physical examination-indicated cerclage compared to their ultrasound-indicate cerclage counterparts.

## 6. Conclusions

Based on the results of this study, the authors suggest that providers consider indication for cerclage before scheduling elective cerclage removal. Women undergoing a non-elective cerclage placement should be aware that they are at risk to require unscheduled cerclage removal before 36 weeks gestational age. Conversely, women who had an elective cerclage (history-indicated) will most likely not require unscheduled cerclage removal before 36 weeks. Women who had a physical examination-indicated cerclage are most likely to deliver within 72 hours of cerclage removal. For women with elective cerclage placement whose cerclage is electively removed at ≥36 weeks, there is no difference in the time interval between removal and delivery regardless of whether cerclage was removed at 36 or 37 weeks gestational age.

## Figures and Tables

**Table 1:** Comparison of maternal demographic data and outcome variables in women treated with elective versus non-elective cerclage.

	Elective cerclage placement (*n* = 39)	Non-elective cerclage placement (*n* = 33)	*p* value
Race Caucasian African American Hispanic	23 (59%) 14 (36%) 2 (5%)	18 (55%) 14 (42%) 1 (3%)	0.85
Age (years)	31.80 ± 0.85	31.28 ± 1.09	0.71
Gravidity	4.13 ± 0.27	4.12 ± 0.40	0.90
Tobacco use	9 (23%)	6 (18%)	0.83
Marijuana use	6 (15%)	1 (3%)	0.12
Prior cervical treatment	17 (44%)	5 (15%)	0.02
Prior spontaneous preterm delivery	36 (92%)	20 (61%)	0.003
GA at cerclage placement (weeks)	15.02 ± 0.34	20.51 ± 0.43	<0.01
Elective cerclage removal at ≥36 weeks	28(71.8%)	13(39.4%)	0.01
GA at cerclage removal (weeks)	34.72 ± 0.81	33.72 ± 0.71	0.36
GA at delivery (weeks)	35.92 ± 0.88	34.72 ± 0.82	0.33
Delivery at ≤72 hour following removal of cerclage	18 (46%)	19 (58%)	0.47
Time interval between removal of cerclage and delivery (days)	8.33 ± 1.67	6.91 ± 1.59	0.64

**Table 2:** Comparison of outcomes between women treated with physical examination-indicated cerclage versus ultrasound indicated cerclage.

	Physical examination indicated cerclage *n* = 15	Ultrasound indicated cerclage *n* = 18	*p* value
GA at cerclage placement (weeks)	21.33 ± 0.37	19.83 ± 0.69	0.07
GA at cerclage removal (weeks)	33.83 ± 0.86	33.62 ± 1.11	0.88
GA at delivery	34.38 ± 0.97	34.99 ± 1.29	0.71
Non-elective removal	7(47%)	7(39%)	0.78
Delivery ≤72 hours following removal of cerclage	12(80%)	7(39%)	0.04
Time interval between removal of cerclage and delivery (days)	3.67 ± 1.86	9.61 ± 2.33	0.14

**Table 3:** Sub-analysis of women who underwent elective cerclage placement, comparing elective cerclage removal at 36 weeks versus those removed at 37 weeks.

	Cerclage removed at 36 weeks *N* = 16	Cerclage removed at 37 weeks *N* = 12	*p* value
Gestational age at cerclage placement (weeks)	14.95 (±0.40)	15.37 (±0.82)	0.65
Delivery within 72 hours of removal of cerclage	5 (31%)	7 (58%)	0.30
Time interval between removal of cerclage and delivery (days)	9.38 (±2.12)	3.83 (±1.43)	0.13

## Data Availability

The data that support the findings of this study are available on request from the corresponding author. The data are not publicly available due to restrictions, e.g., their containing information that could compromise the privacy of research participants.
